# An experimental investigation into the borehole drilling and strata characteristics

**DOI:** 10.1371/journal.pone.0253663

**Published:** 2021-07-20

**Authors:** Cancan Liu, Xigui Zheng, Niaz Muhammad Shahani, Peng Li, Cong Wang, Xiaowei Guo

**Affiliations:** Key Laboratory of Deep Coal Resource Mining, Ministry of Education of China, School of Mines, China University of Mining and Technology, Xuzhou, China; RMIT University, AUSTRALIA

## Abstract

Measurement while drilling is an important part of the intelligent development of coal mines. The main purpose of this paper is to comprehensively analyze the response characteristics of borehole drilling parameters and find a better method to predict rock mechanical properties based on drilling parameters. Firstly, six concrete blocks and multiple specimens were prepared with different material ratios. Next, the concrete specimens were tested for mechanical properties in the laboratory. Meanwhile, the displacement, rotation speed, torque, and sound pressure level (SPL) were observed during the drilling of the concrete blocks. Finally, the response characteristics of drilling parameters such as rotation speed, rate of penetration (ROP), torque, and SPL were analyzed. Besides, multiple prediction models of rock mechanical parameters were obtained by data analysis. The research results indicate that the drilling process can be classified into the initial stage of drilling (fast speed) and the steady stage of drilling (slow speed). The torque work ratio accounts for more than 99%, which increases with the increase in rock strength. The penetration depth per revolution and torque work ratio are significantly related to rock uniaxial compressive strength, Brazilian tensile strength, cohesion, and elastic modulus. The ROP is the best choice for estimating rock mechanical parameters. This research provides an important reference for laboratory rock mechanics parameter testing and geological features detection based on drilling parameters.

## Introduction

Rock formation characteristics are regarded as an important basis for the selection of support schemes and design parameters in coal mines. However, the traditional rock coring method has disadvantages such as slow speed, high cost, and high labor intensity, which can no longer meet the development trend of intelligent construction of coal mines [1]. At present, comprehensive data analysis shows that drilling parameters can be employed to estimate rock mass strength [2]. Measurement while drilling (MWD) is an important part of precision coal mining and has become a current research hotspot.

**Fig 1:**
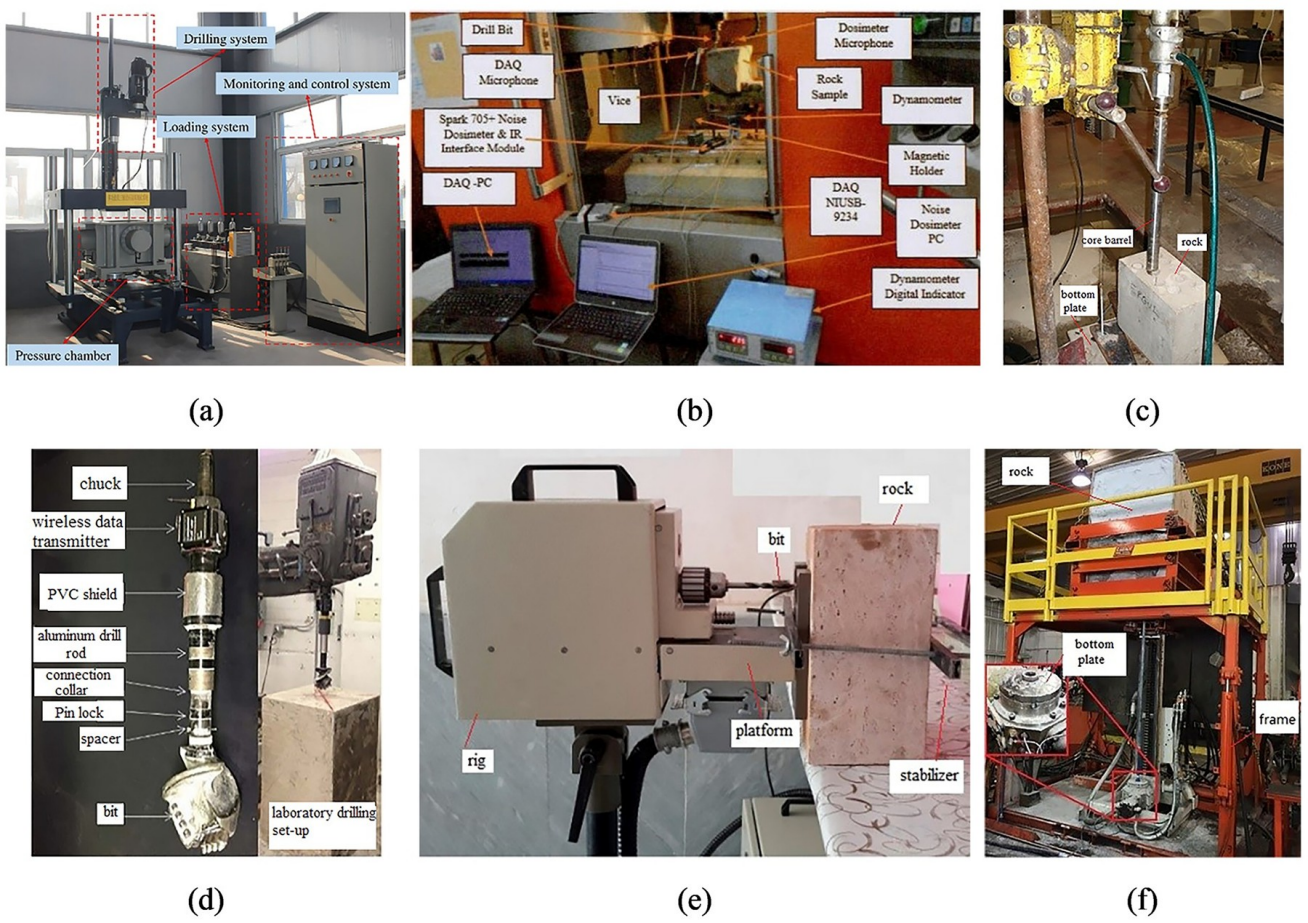
The main MWD devices developed in the laboratory.

At present, there are several MWD devices developed for laboratory testing. For example, the prediction models of rock uniaxial compressive strength (UCS), cohesion, and internal friction angle was established based on the drilling parameters obtained through the multifunctional rock drilling test system shown in Fig 1(a) [3, 4]. Kumar et al. established prediction models of UCS, Brazilian tensile strength (BTS), and porosity based on sound pressure level (SPL) obtained by the equipment shown in Fig 1(b) [5]. This device is used to drill rock by setting a constant rotation speed and rate of penetration (ROP). Only SPL can be recorded during drilling. Similarly, rotation speed, ROP, SPL and drill bit diameter were used as input parameters in the artificial neural network to predict UCS, BTS, modulus of elasticity, Schmidt rebound number, dry density, P-wave velocity, and percentage porosity [6]. The dominant sound signal frequencies were also used to predict rock UCS, BTS, and density [7]. Yaşar et al. found that the ROP gradually decreases as the drilling depth and time increase based on the MWD device shown in Fig 1(c) [8]. Rodgers et al. conducted rock drilling experiments in the laboratory through the device shown in Fig 1(d) and found out that torque and UCS have a higher correlation than thrust and UCS [9]. Additionally, the relationship model between mechanical specific energy (MSE) and UCS was established to predict the drilled formation strength [10]. Lakshminarayana et al. established the prediction models of rock UCS, BTS based on the drill bit diameter, rotation speed, ROP, thrust, and vibration frequency [11]. A portable drilling machine was developed to drill rock, as shown in Fig 1(e), and the obtained parameters were used to estimate UCS, cohesion and internal friction angle [12]. Vardhan et al. found a significant correlation between SPL and rock mechanical properties based on a specially developed percussion drill setup [13]. Based on the J.H. Fletcher drilling system, shown in Fig 1(f), Rostami et al. studied the vibration response characteristics of the drill bit passing through a rock fissure during rock drilling [14]. The composite index established based on the pattern recognition algorithm with the thrust, torque, and ROP greatly reduces the false alarm rate of fissure recognition [15, 16]. Numerical simulation is also used to simulate the drilling process. For instance, the rock cutting process with roadheader picks was simulated with the discrete element method [17].

In this article, an attempt has been made to extend the application of MWD to predict the mechanical properties of roadway roof strata. In coal mines, borehole drilling is an integral part of the working cycle. From previous research, it is found that most of the equipment drills down into the rock, which is more suitable for simulating drilling in petroleum reservoirs. Furthermore, most of the experimental methods use preset drilling parameters to simulate rock drilling such as preset rotation speed and ROP, measuring torque, and thrust, or preset thrust and torque, measuring rotation speed and ROP. Nevertheless, borehole drilling is an adaptive system in coal mines. The drilling parameters all change accordingly when the drill bit encounters different rock formations [18]. At present, many scholars have studied the relationship between drilling parameters and rock mechanical properties in the laboratory [19, 20]. However, the response characteristics of borehole drilling parameters and the relationship with rock mechanical properties have not been fully analyzed.

To address these problems, based on the developed MWD device, 6 concrete blocks of different strengths were poured to replace different strata. Meanwhile, the mechanical properties of each concrete specimen were determined in the laboratory. Then, the correlation between the borehole drilling parameters and UCS, BTS, cohesion, internal friction angle, elastic modulus, and Poisson’s ratio was comprehensively analyzed. Finally, the obtained prediction models of various rock mechanics parameters were compared. This research provides a reference for laboratory rock mechanics parameter testing and roadway roof rock formation detection in coal mines.

## Experimental equipment and materials

### Equipment

**Fig 2:**
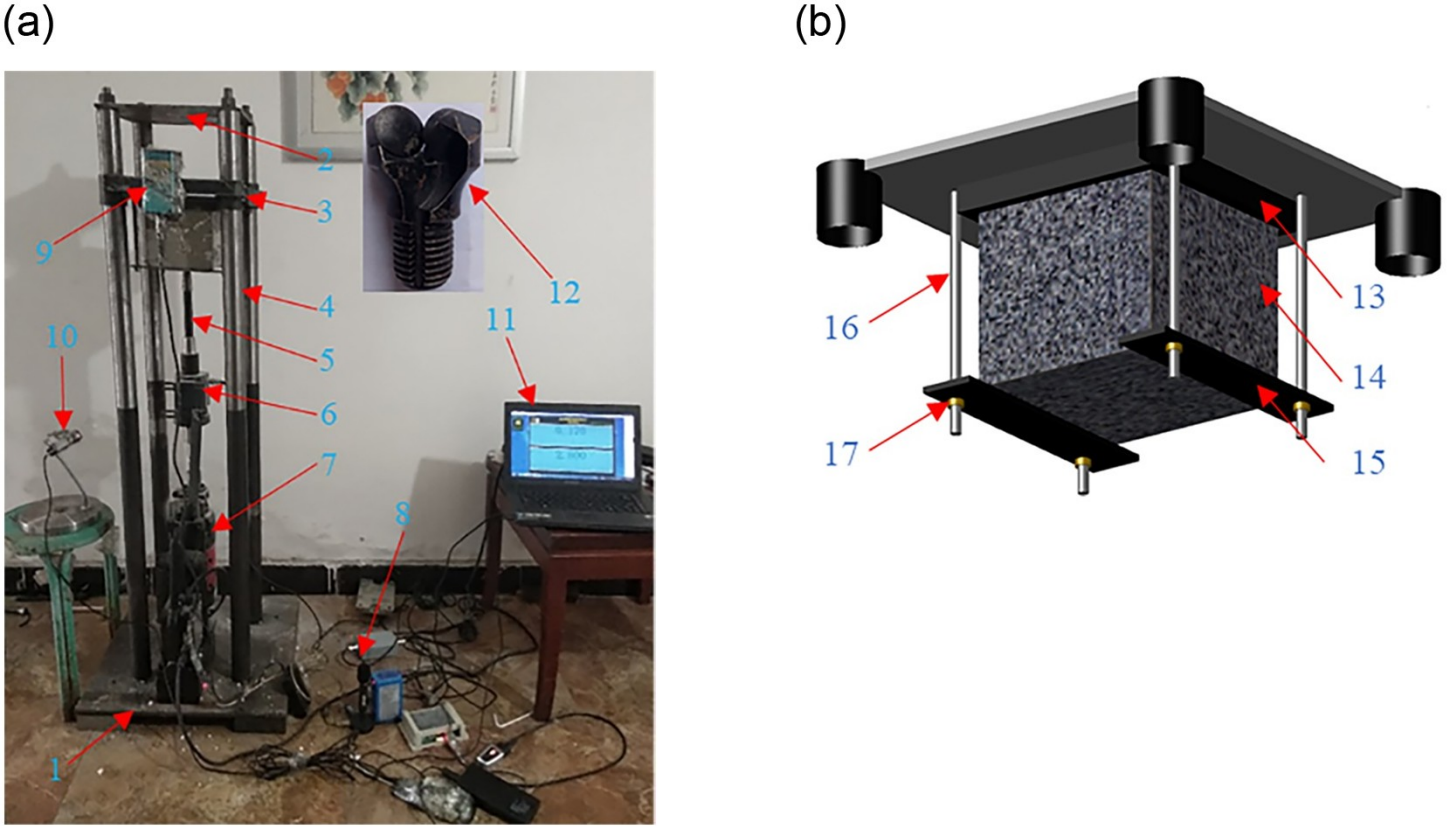
Simulation device for drilling boreholes. 1-Base plate; 2-Upper fixing plate; 3- Gravity loading plate; 4-Guiding column; 5-Drill rod; 6-Torque sensor; 7-Electric drill; 8- SPL recorder; 9-Displacement sensor; 10-Rotation speed sensor; 11-Computer; 12-Drill bit; 13-Groove; 14-Rock sample; 15-Fixed plate; 16-Fixed column; 17-Nut. [21].

As shown in Fig 2(a), the self-designed device was used to simulate borehole drilling in the laboratory [21]. The size of the base plate is length×width×height = 500×500×60mm. The length and diameter of the 4 guide columns are 1350mm and 45mm, respectively. The size of the upper fixing plate is length×width×height = 300×300×8mm. The gravity loading plate in the middle can move up and down along the guide column through the guide sleeve. The diameter and height of the guide sleeve are 46mm and 50mm, respectively. Gravity can be applied to the gravity loading plate to simulate the thrust of the anchor rig. The drill bit is a two-wing diamond composite chip drill bit with a diameter of 28mm, which is suitable for rock formations with a Platts coefficient less than 10, and is mainly used for drilling boreholes. The rock sample with the size of 150×150×150mm is fixed under the gravity loading plate according to the method shown in Fig 2(b). The groove size is 152×152×20mm, which is used to fix the top of the sample. The bottom of the sample is fixed with the fixed column, fixed plate and nut.

### Materials

The detachable mold with a size of 150mm×150mm×150mm was used to pour concrete blocks. Cylindrical molds with a diameter of 50mm and a height of 100mm, 50mm, and 25mm were used to process the test specimens for physical and mechanical properties. Using sand (particle size less than 3mm), loess, 42.5 composite Portland cement, 32.5 slag Portland cement, and water as raw materials, 6 different strength concrete blocks and test pieces were poured according to the ratio shown in Table 1. To ensure that at least 3 valid data are obtained for each group of specimens, 18 specimens of each type of concrete were poured, including 6 UCS specimens, 6 BTS specimens and 6 shear specimens.

**Table 1:** Material ratio of different strength concrete blocks (kg/m^3^).

Number	sand	loess	42.5 composite Portland cement	32.5 slag Portland cement	water
1	400	1259.26	0	711.11	444.44
2	533.33	453.33	0	1422.22	
3	800	0	0	1659.26	
4	800	0	1659.26	0	
5	0	1007.41	0	1422.22	
6	533.33	755.56	1185.19	0	

The concrete was mixed for the same amount of time before being loaded into the molds and vibrated for the same amount of time after being loaded into the molds to ensure consistency throughout the specimens. These concrete samples were cured for 180 days under the same conditions. Finally, the UCS, BTS, and shear specimens were obtained by cutting machine and stone grinding machine.

## Experimental method

### Mechanical properties test of concrete specimens

Firstly, all samples were numbered according to the following rules. The UCS, BTS, and shear specimens were denoted by A, B, and C, respectively. The number A1-2 indicates the No. 2 specimen of the No. 1 concrete block for the UCS test. Next, the diameter and height of all test pieces were determined by the average of 3 measurements with an electronic vernier caliper. Finally, the mass of each specimen was measured by an electronic scale to calculate the density of each concrete block.

A set of strain gauges were attached to the symmetry plane of each UCS specimen to measure the Poisson’s ratio of various specimens. The BX120-20AA strain gauge was arranged transversely in the middle of the specimen, and the BX120-50AA strain gauge was arranged longitudinally on the specimen next to the transverse strain gauge.

The angles of the designed shear experiment are 45°, 50°, 55°, and 60°. TAJ-1000 electro-hydraulic servo rock testing machine was used for physical and mechanical testing, and the loading rate was set to 0.5MPa/s.

### Simulation of drilling boreholes

**Fig 3:**
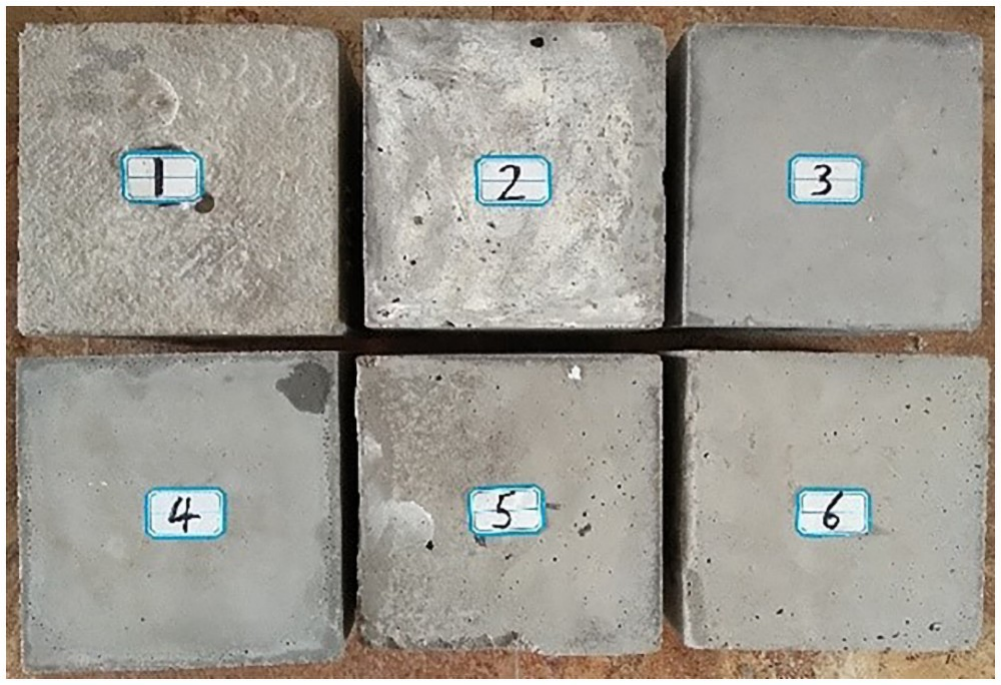
Concrete blocks of different strengths.

Fig 3 shows concrete blocks of different strengths being poured. Using the constant gravity (thrust) to drill different concrete blocks is inconsistent with the actual situation. It can be known from the existing research work that the thrust of the anchor rig increases as the rock formation strength increases [22]. Therefore, the gravity difference of each concrete block was used to represent the thrust difference during drilling. In this way, the thrust, torque, ROP, and rotation speed can be automatically adjusted while drilling concrete blocks with different strengths, which is closer to the actual situation. Drilling was turned off the power at the drilling depth reaches 50mm.

The drilling of boreholes was performed as follows: i. The total gravity of the gravity-loaded plate and the concrete block, which is regarded as the thrust during drilling. ii. The concrete block is fixed under the gravity loading plate, and lubricating oil is evenly spread on the guide column to minimize the impact of resistance during sliding. iii. The position of the rig is adjusted so that the drill bit is aligned with the center of the concrete block, and the magnetic is switched on to make the bottom of the rig attached to the base plate. iv. The rotation speed sensor, displacement sensor, torque sensor and SPL recorder are connected, and each of them is debugged through the computer to ensure that they are in a normal state. v. Drilling of boreholes stars and the data is recorded in real-time.

## Results and analysis

### Test results of concrete mechanical properties

After sample processing and testing, the effective data obtained by each group is as follows: For the UCS test, 3 effective data were obtained for No. 1 concrete, and 4 effective data were obtained for other concretes. For the BTS test, 3 valid data were obtained for No. 1 concrete, and 6 valid data were obtained for other concretes. For the shear test, 3 and 6 valid data were obtained for No. 1 and No. 4 concretes respectively, and 4 valid data were obtained for other concretes. The average values of the UCS, BTS, elastic modulus and Poisson’s ratio of each concrete block were taken as the physical and mechanical parameters of the concrete specimen. Table 2 presents the physical and mechanical parameters of each concrete specimen.

**Table 2:** Test results of physical and mechanical properties of concrete specimens.

Number	Density (g/cm^3^)	UCS (MPa)	BTS (MPa)	*C* (MPa)	*φ* (°)	*μ*	*E* (MPa)
1	1.542	0.30	0.04	0.26	26.27	0.37	14.96
2	1.991	5.59	0.42	2.60	15.68	0.25	675.69
3	2.023	15.85	0.93	4.16	31.65	0.22	1490.53
4	2.049	25.90	1.23	5.71	27.83	0.20	1962.23
5	1.901	5.56	0.30	1.74	29.83	0.30	744.56
6	1.927	6.76	0.55	1.99	33.52	0.26	1048.06

### The relationship between borehole drilling parameters and concrete mechanical properties

#### The relationship between ROP and concrete mechanical properties

**Fig 4:**
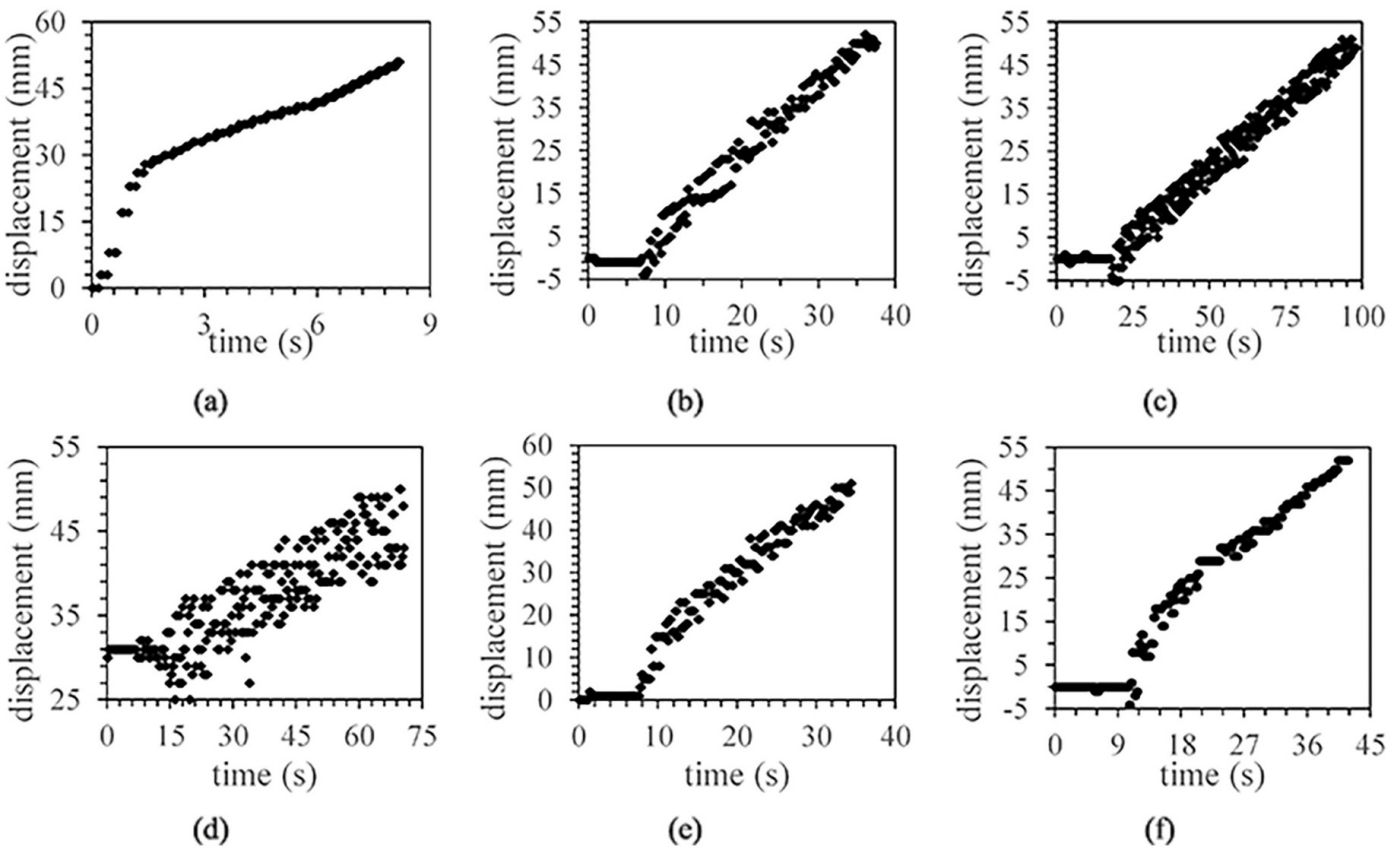
Displacement data distribution characteristics of each concrete block during drilling. (a)No. 1 concrete. (b)No. 2 concrete. (c)No. 3 concrete. (d)No. 4 concrete. (e)No. 5 concrete. (f)No. 6 concrete.

Fig 4(d) shows the displacement data of the 30-50mm section of the borehole due to invalid data collected for the 0-30mm section. From the complete drilling process, the ROP is relatively fast during the initial period and then slows down. Therefore, the drilling process can be divided into the initial phase and the stabilization phase. The initial stage range of concrete with different strength is different, but it is mainly concentrated in 0-30mm. In addition, it can be found that the displacement data during drilling is not in a straight line due to vibration. If the linear model is used to fit the data of the drilling stability stage and the offset fitting curve distance is used to evaluate the fluctuation amplitude which tends to increase with the increase of rock strength.

The following work was done to study the relationship between ROP and concrete mechanical parameters. Firstly, the average ROP at the drilling stable stage was regarded as the ROP of each specimen. The stabilized ROP for each specimen is displayed in Table 3.

**Table 3:** The stabilized ROP for each specimen.

Sample No.	1	2	3	5	5	6
Stabilized ROP (mm/s)	3.23	1.72	0.58	0.29	1.34	1.25

**Fig 5:**
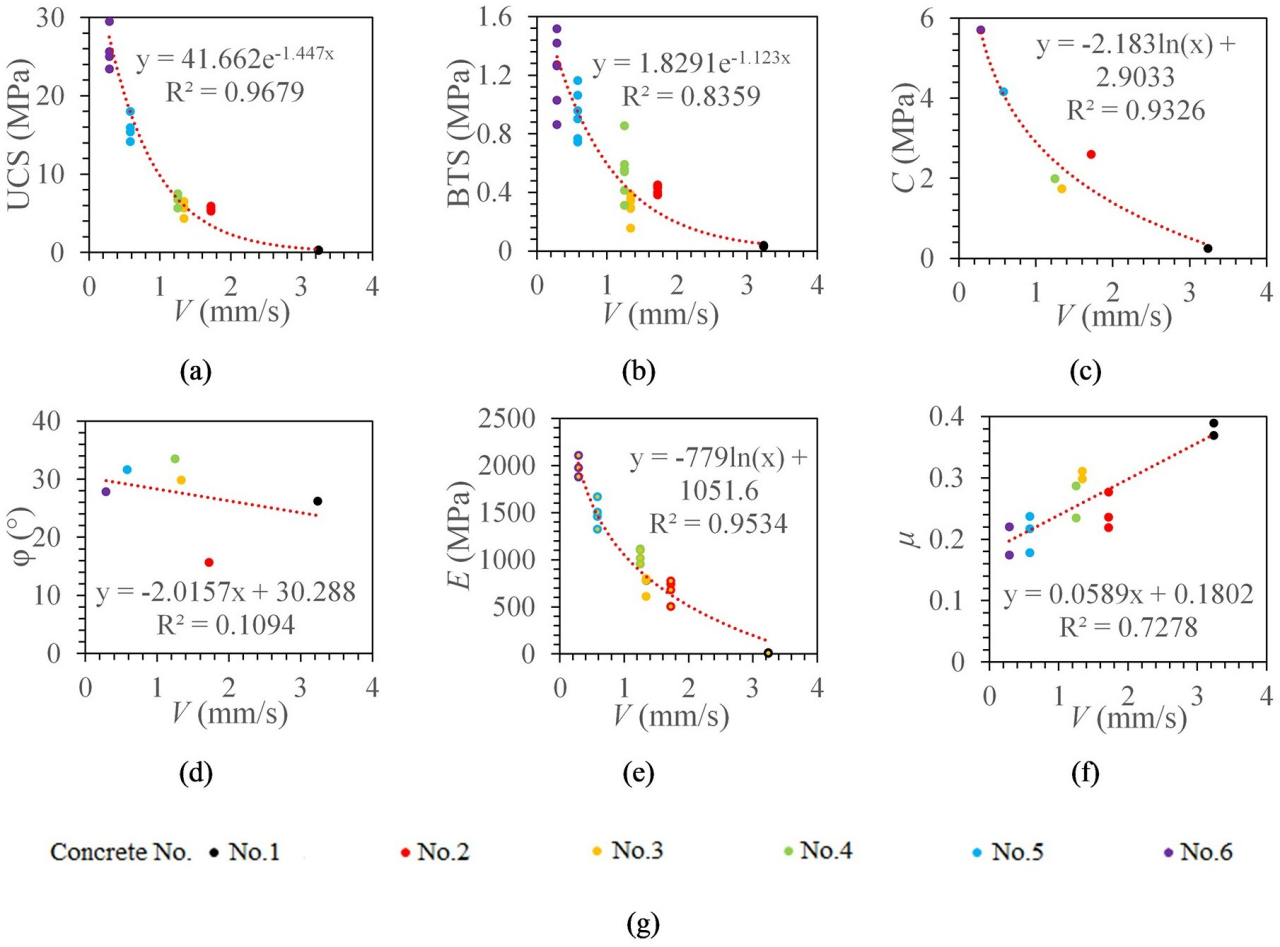
The relationship between the stabilized ROP and the concrete mechanical parameters. (a) The relationship between *V* and UCS. (b) The relationship between *V* and BTS. (c) The relationship between *V* and *C*. (d) The relationship between *V* and *φ*. (e) The relationship between *V* and *E*. (f) The relationship between *V* and *μ*. (g) legend.

Then, the exponential function, power function, logarithmic function, and linear function were respectively used to fit the ROP and UCS to obtain the optimal fitting function. Similarly, the optimal fitting functions of ROP and BTS, cohesion, internal friction angle, elastic modulus, and Poisson’s ratio can be obtained by the above method. Fig 5 shows the best-fitting curves between the stabilized ROP and the concrete mechanical parameters.

It can be seen from Fig 5, the ROP has an exponential relationship model with UCS and BTS, and the correlation coefficients are 0.9631 and 0.8718, respectively. The ROP has a logarithmic relationship model with cohesion and elastic modulus, and the correlation coefficients are 0.9326 and 0.9534, respectively. The ROP has a linear relationship with Poisson’s ratio (R^2^ = 0.7278) and has no significant correlation with the internal friction angle (R^2^ = 0.1094). When the ROP is higher, the UCS, BTS, cohesion and elastic modulus of the specimen are smaller, and the Poisson’s ratio is larger. The ROP has the highest correlation with UCS.

#### The relationship between SPL and concrete mechanical properties

**Fig 6:**
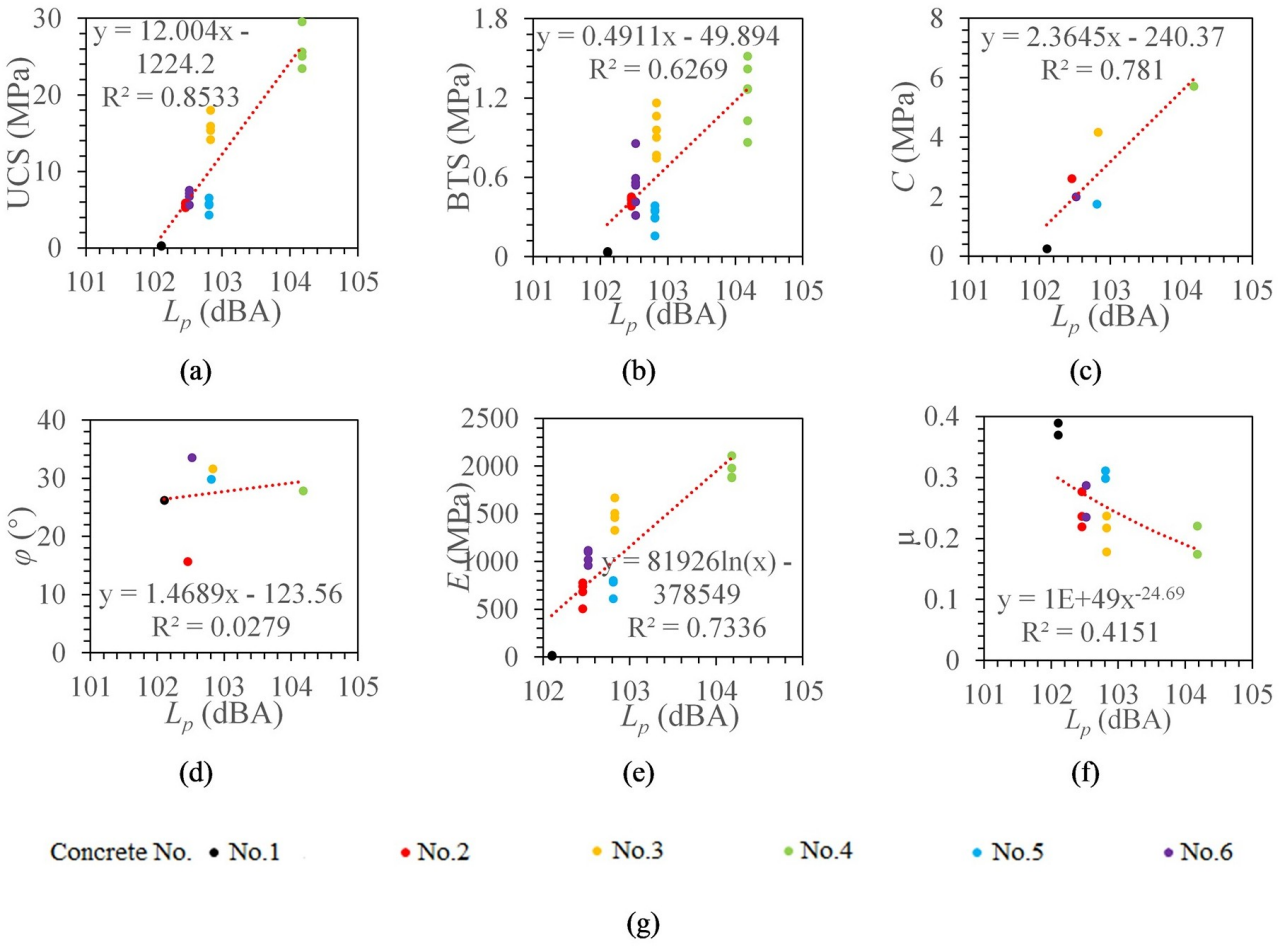
The relationship between SPL and concrete mechanical properties. *L*_*p*_ signifies SPL. (a) The relationship between *L*_*p*_ and UCS. (b) The relationship between *L*_*p*_ and BTS. (c)The relationship between *L*_*p*_ and *C*. (d) The relationship between *L*_*p*_ and *φ*. (e) The relationship between *L*_*p*_ and *E*. (f) The relationship between *L*_*p*_ and *μ*. (g) legend.

The average value of the SPL during drilling of the 30-50mm section was taken as the SPL of the concrete. Specimen No. 5 has the highest SPL (104.2dBA), while specimen No. 1 has the lowest SPL level (102.1dBA). The SPL tends to increase when the strength of the drilled specimen increases. To further analyze the relationship between SPL and concrete mechanical parameters, the same data fitting method described above was reused here. Fig 6 shows the best fitting models between the SPL and the concrete mechanical parameters.

It can be seen from Fig 6 that the SPL has a linear relationship with UCS, BTS, and cohesion, and their correlation coefficients are 0.8533, 0.6269, and 0.781, respectively. SPL and elastic modulus are in a logarithmic function model, and the correlation coefficient is 0.7336. SPL has no significant correlation with the internal friction angle and Poisson’s ratio, and the correlation coefficients are 0.0279 and 0.3948, respectively. SPL has the greatest correlation with UCS.

#### The relationship between torque and concrete mechanical properties

**Fig 7:**
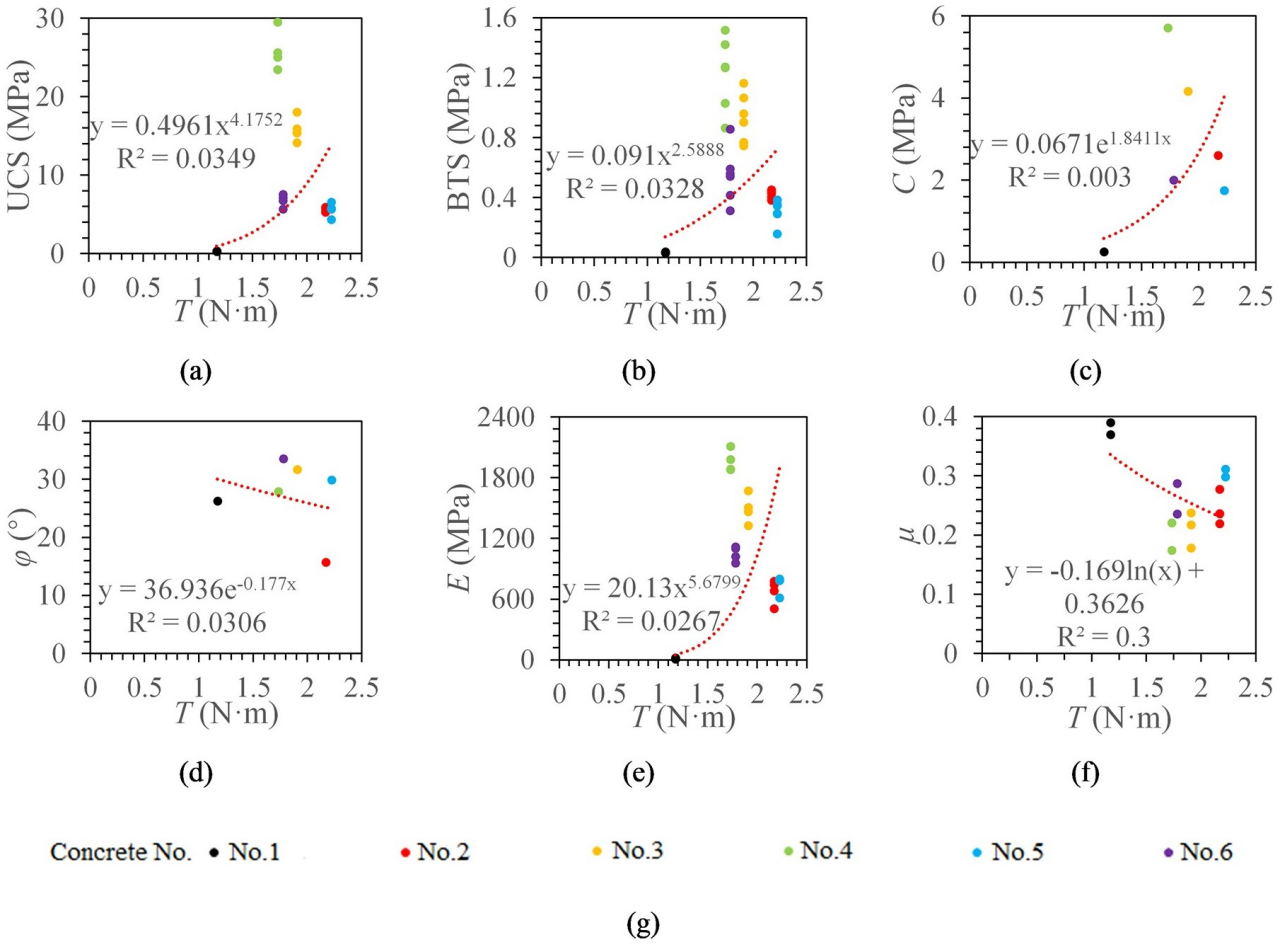
The relationship between torque and the concrete mechanical properties. *T* indicates torque. (a) The relationship between *T* and UCS. (b) The relationship between *T* and BTS. (c) The relationship between *T* and *C*. (d) The relationship between *T* and *φ*. (e) The relationship between *T* and *E*. (f) The relationship between *T* and *μ*. (g) legend.

The same data fitting method described above was reused here. Fig 7 shows the best fitting models between the torque and the concrete mechanical parameters.

It can be seen from Fig 7 that the significant coefficients of torque and UCS, BTS, cohesion, internal friction angle, elastic modulus, and Poisson’s ratio are all less than 0.6. It can be found that the torque increases first and then decreases with the UCS increase. Therefore, there may be a critical UCS value *R*_*i*_. The torque decreases with the increase of rock strength when the UCS is greater than *R*_*i*_; The torque decreases with the decrease of rock strength when the UCS is less than *R*_*i*_.

#### The relationship between rotation speed and concrete mechanical properties

The average rotation speed in the stable stage was taken as the rotation speed of the specimen. The optimal fitting model of rotation speed and UCS, BTS, cohesion, internal friction angle, elastic modulus, and Poisson’s ratio can be obtained by using the same method mentioned above.

**Fig 8:**
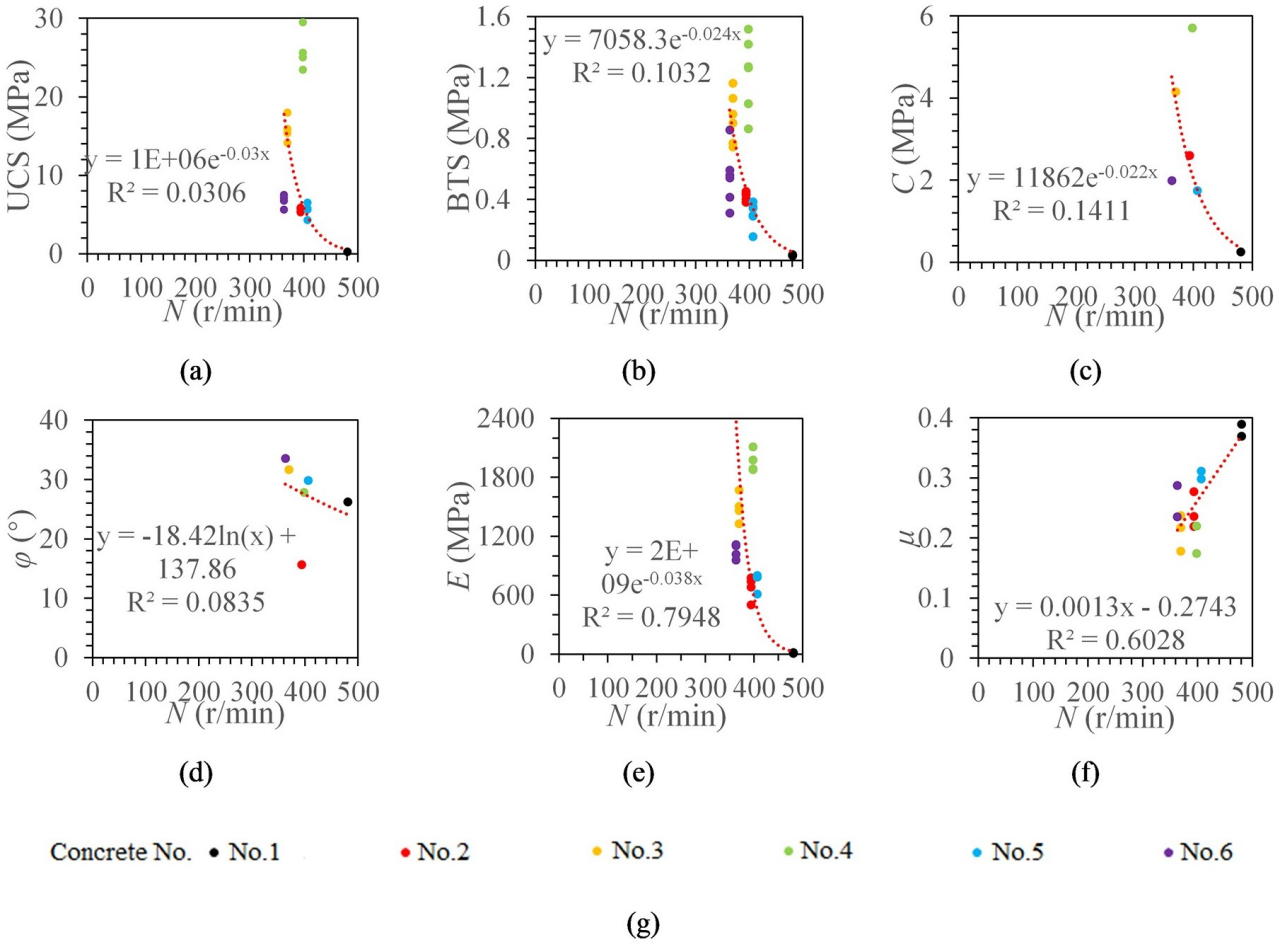
The relationship between rotation speed and concrete mechanical properties. ***N* denotes rotation speed**. (a) The relationship between *N* and UCS. (b) The relationship between *N* and BTS. (c) The relationship between *N* and *C*. (d) The relationship between *N* and *φ*. (e) The relationship between *N* and *E*. (f) The relationship between *N* and *μ*. (g) legend.

It can be seen from Fig 8 that the rotation speed and elastic modulus are in an exponential function model with the highest correlation (R^2^ = 0.7948). The rotation speed and UCS, BTS and cohesion are all exponential function models, and the correlation coefficients are mainly concentrated in the range 0.6–0.7. The rotation speed has a linear relationship with Poisson’s ratio, and the correlation coefficient is 0.6028. There is no significant correlation between rotation speed and internal friction angle (R^2^ = 0.0835). Also, it can be found that the rotation speed tends to first decrease and then increase when the UCS gradually increases.

#### The relationship between penetration depth per revolution and concrete mechanical properties

The penetration depth per revolution varies with rocks of different strengths. Theoretically, when the strength of the rock formation is higher, the penetration depth per revolution becomes smaller. The penetration depth per revolution can be described as:

$$h=\frac{60*V}{N}$$ (1)

Where *h* represents penetration depth per revolution, mm/r, *V* indicates ROP, mm/s, *N* signifies rotation speed, r/min.

**Fig 9:**
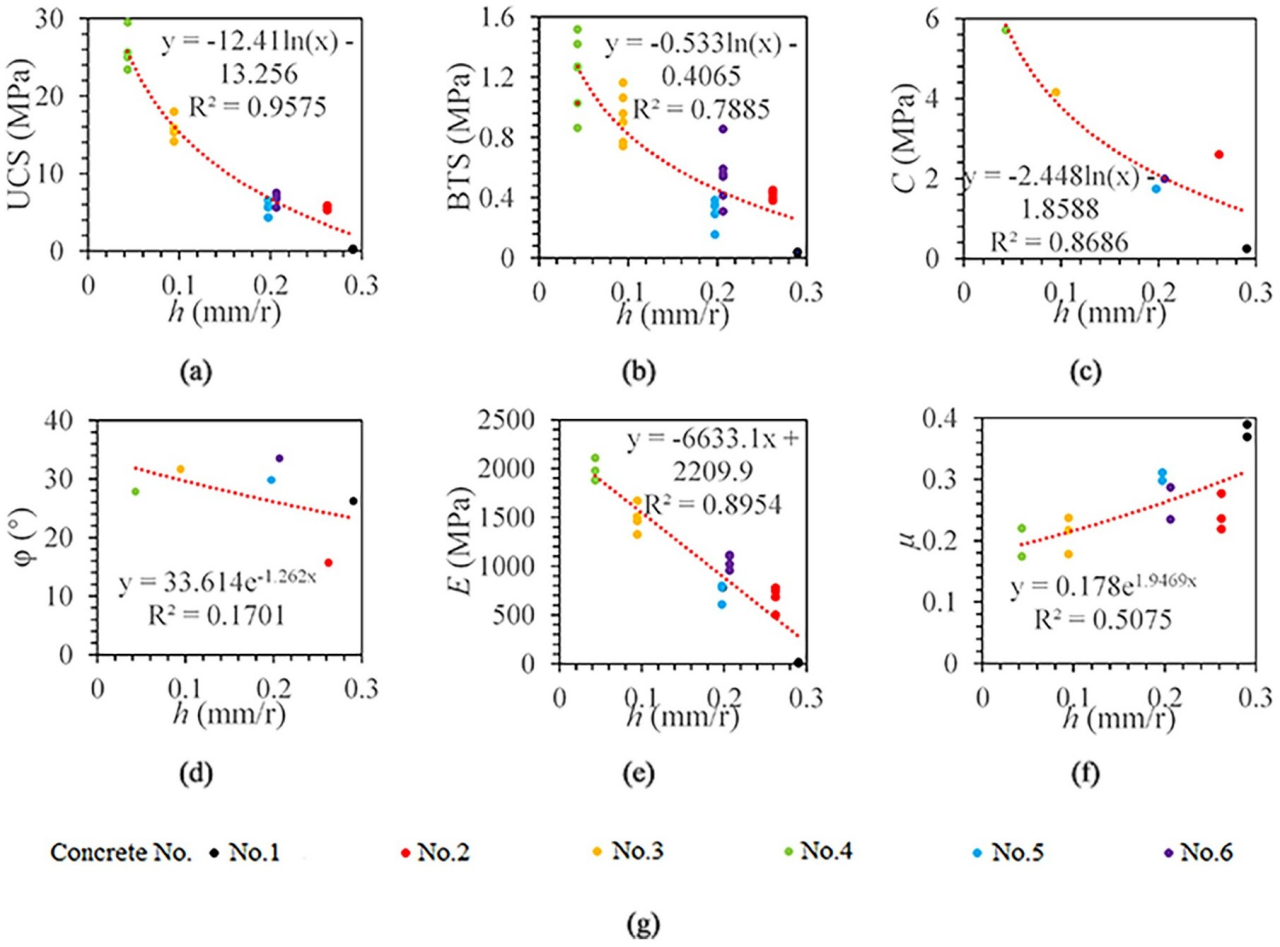
The relationship between penetration depth per revolution and concrete mechanical properties. (a) The relationship between *h* and UCS. (b) The relationship between *h* and BTS. (c) The relationship between *h* and *C*. (d) The relationship between *h* and *φ*. (e) The relationship between *h* and *E*. (f) The relationship between *h* and *μ*. (g) legend.

To further analyze the relationship between penetration depth per revolution and concrete mechanical parameters, the same data fitting method described above was reused here. Fig 9 shows the best fitting models between penetration depth per revolution and the concrete mechanical parameters.

It can be seen from Fig 9 that with the increase in penetration depth per revolution, the UCS, BTS, cohesion, and elastic modulus gradually decrease, and the Poisson’s ratio has a gradually increasing trend. The penetration depth per revolution and UCS, BTS, and cohesion are logarithmic function models, and the correlation coefficients are 0.9575, 0.7885, and 0.8686, respectively. The penetration depth per revolution has a linear relationship with the elastic modulus, and the correlation coefficient is 0.8954. The penetration depth per revolution and Poisson’s ratio are in an exponential function model, and the correlation coefficient is 0.5231. There is no significant correlation between the penetration depth per revolution and the internal friction angle (R^2^ = 0.1932).

### The relationship between work done and mechanical parameters during drilling

#### Theoretical calculation of energy during drilling

**Fig 10:**
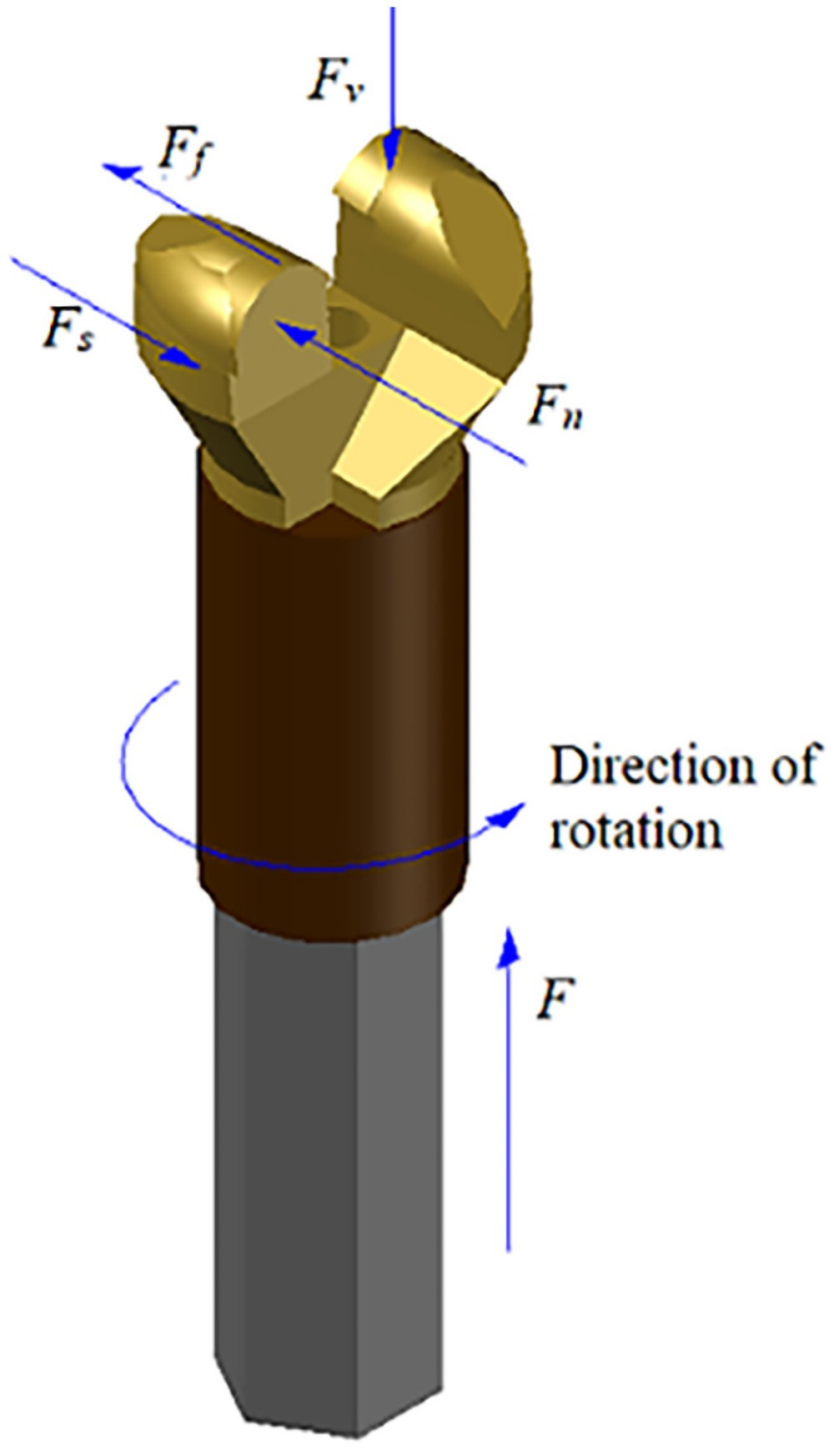
Mechanical model of drill bit.

The mechanical model of the bit is shown in Fig 10. While drilling vertically upwards with thrust *F* and torque *T*, the bit top is subjected to reaction force *F*_*V*_ and friction force *F*_*f*_. *F*_*s*_ is the cutting force generated by the torque. *F*_*n*_ is the resistance when the rock is broken under the action of rotation.

The drill bit mainly intrudes into the rock by thrust and then breaks the rock under the action of rotation. Hence, work is mainly done by thrust and torque during the drilling process. Teale (1965) [23] proposed the MSE model and calculated the thrust work and torque work required to drill a unit volume of rock using the following formulas:

$$W_{F}=\frac{F}{\pi r^{2}}$$ (2)

$$W_{T}=\frac{TN}{30r^{2}V}$$ (3)

Where *W*_*F*_ and *W*_*T*_ respectively represent thrust work and torque work when drilling a unit volume of rock, N·m. *F* indicates thrust, N. *r* denotes borehole diameter, m. *T* signifies torque, N·m.

It is worth noting that the borehole diameter was replaced by the drill bit diameter in past studies [24, 25]. However, there is an expansion of the borehole diameter indicated by the literature [26]. Therefore, the measured borehole diameter was used to calculate the MSE. Additionally, part of the torque is used to break the rock, and the other part is mainly to overcome the friction between the bit top and the rock [27]. Simplifying the arm of frictional resistance to *r*/2, the frictional work can be expressed as:

$$W_{f}=\frac{fFN}{60rV}$$ (4)

Where *W*_*f*_ denotes friction work, N·m. *f* indicates the friction coefficient between the drill bit and the rock, *f* = 0.21 [28].

According to formulas (2)–(4), the MSE model can be optimized as:

$$MSE=W_{F}+W_{T}-W_{f}$$ (5)

#### Correlation analysis of work and concrete mechanical parameters

The torque work ratio *η* can be solved as follow:

$$\eta =\frac{W_{T}}{W_{F}+W_{T}}\times 100\%$$ (6)

**Fig 11:**
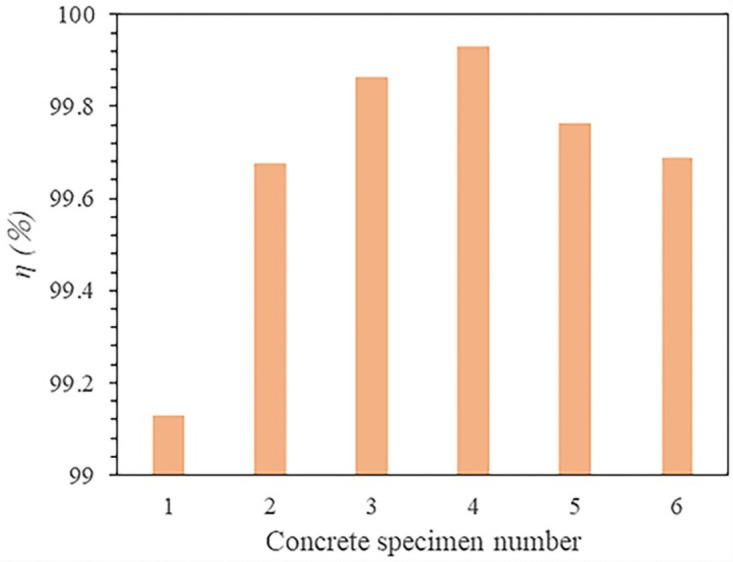
The torque work ratio during the drilling process of concrete specimens with different strengths.

It can be seen from Fig 11 that the torque work ratio accounts for more than 99%, which shows that the specimen is mainly broken by rotating during the drilling process. Moreover, as the strength of the test specimen increases, the torque work ratio gradually increases.

The optimal fitting model of torque work ratio with UCS, BTS, cohesion, internal friction angle, elastic modulus, and Poisson’s ratio were obtained by data fitting.

**Fig 12:**
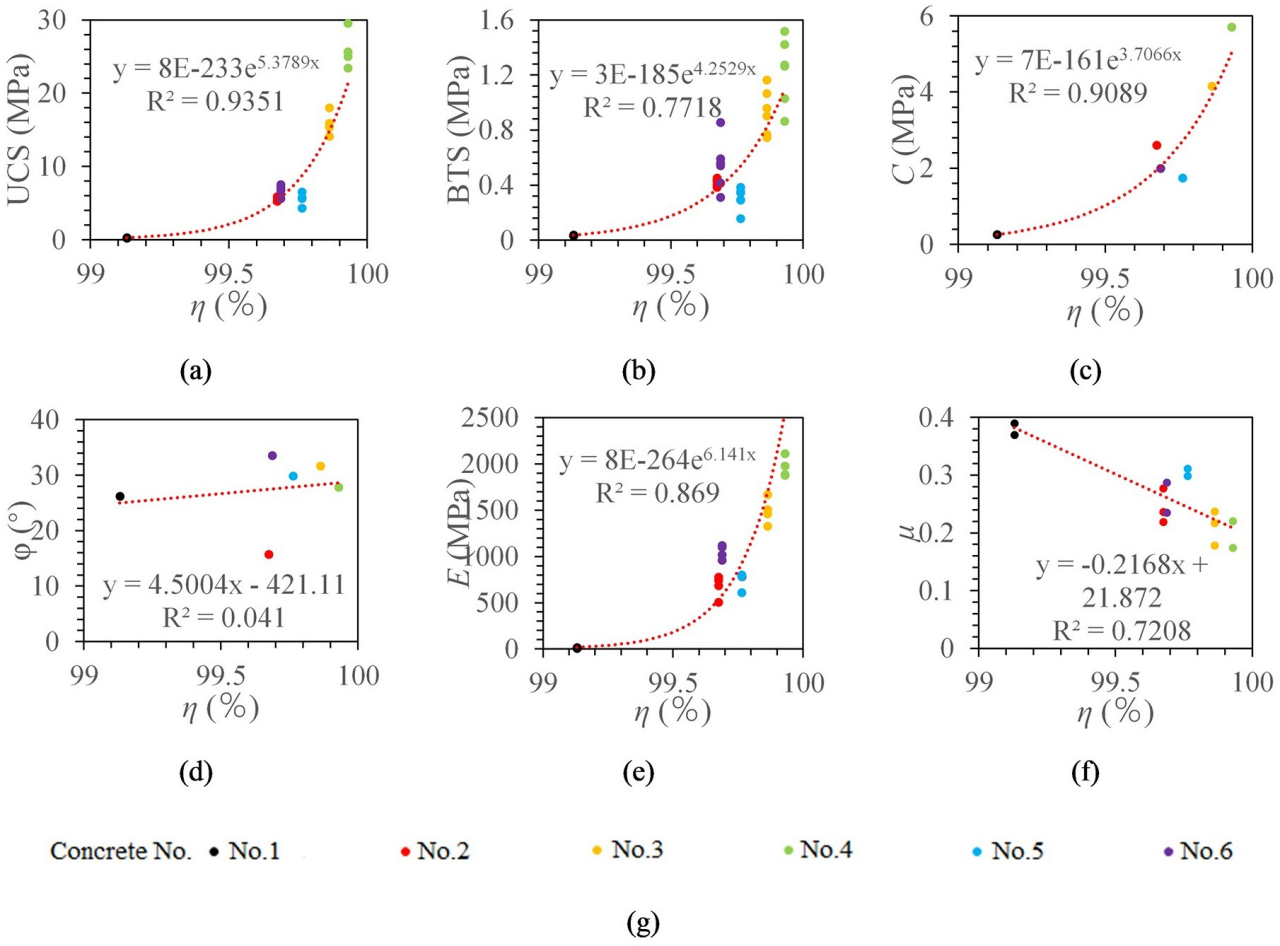
The relationship between torque work ratio and concrete mechanical properties. (a) The relationship between *η* and UCS. (b) The relationship between *η* and BTS. (c) The relationship between *η* and *C*. (d) The relationship between *η* and *φ*. (e) The relationship between *η* and *E*. (f) The relationship between *η* and *μ*. (g) legend.

It can be seen from Fig 12 that the torque work ratio and UCS, BTS, cohesion, and elastic modulus are all exponential function models, and the correlation coefficients are 0.9662, 0.8546, 0.9492, and 0.9479, respectively. The torque work ratio has a linear relationship with Poisson’s ratio, and the correlation coefficient is 0.7208. The torque work ratio has no significant correlation with the internal friction angle (R^2^ = 0.041).

**Fig 13:**
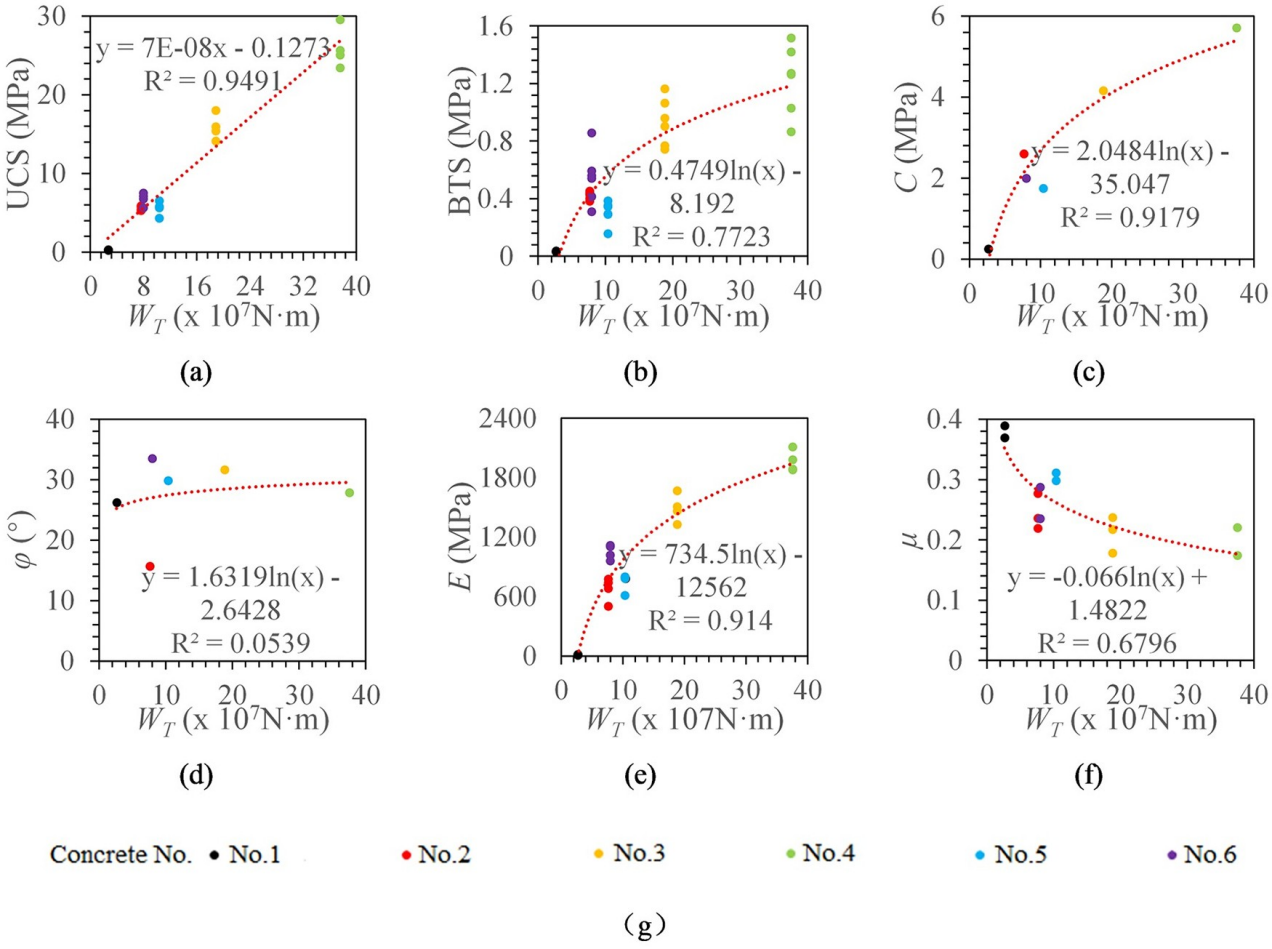
The relationship between torque work and concrete mechanical properties. (a) The relationship between *W*_*T*_ and UCS. (b) The relationship between *W*_*T*_ and BTS. (c) The relationship between *W*_*T*_ and *C*. (d) The relationship between *W*_*T*_ and *φ*. (e) The relationship between *W*_*T*_ and *E*. (f) The relationship between *W*_*T*_ and *μ*. (g) legend.

As shown in Fig 13, the fitting models of torque work and various mechanical parameters were obtained by the above method. The torque work has a linear relationship with the UCS and a logarithmic relationship with BTS, cohesion, elastic modulus, and Poisson’s ratio. Torque work has a significant correlation with UCS, cohesion, and elastic modulus, and the correlation coefficients are all above 0.9. It has the highest correlation with UCS (R^2^ = 0.9491) and has no significant correlation with the internal friction angle. The correlation coefficients between torque work and BTS and Poisson’s ratio are 0.7723 and 0.6796, respectively. Compared with the torque work ratio, it is found that the correlation between torque work and various mechanical parameters is lower.

**Fig 14:**
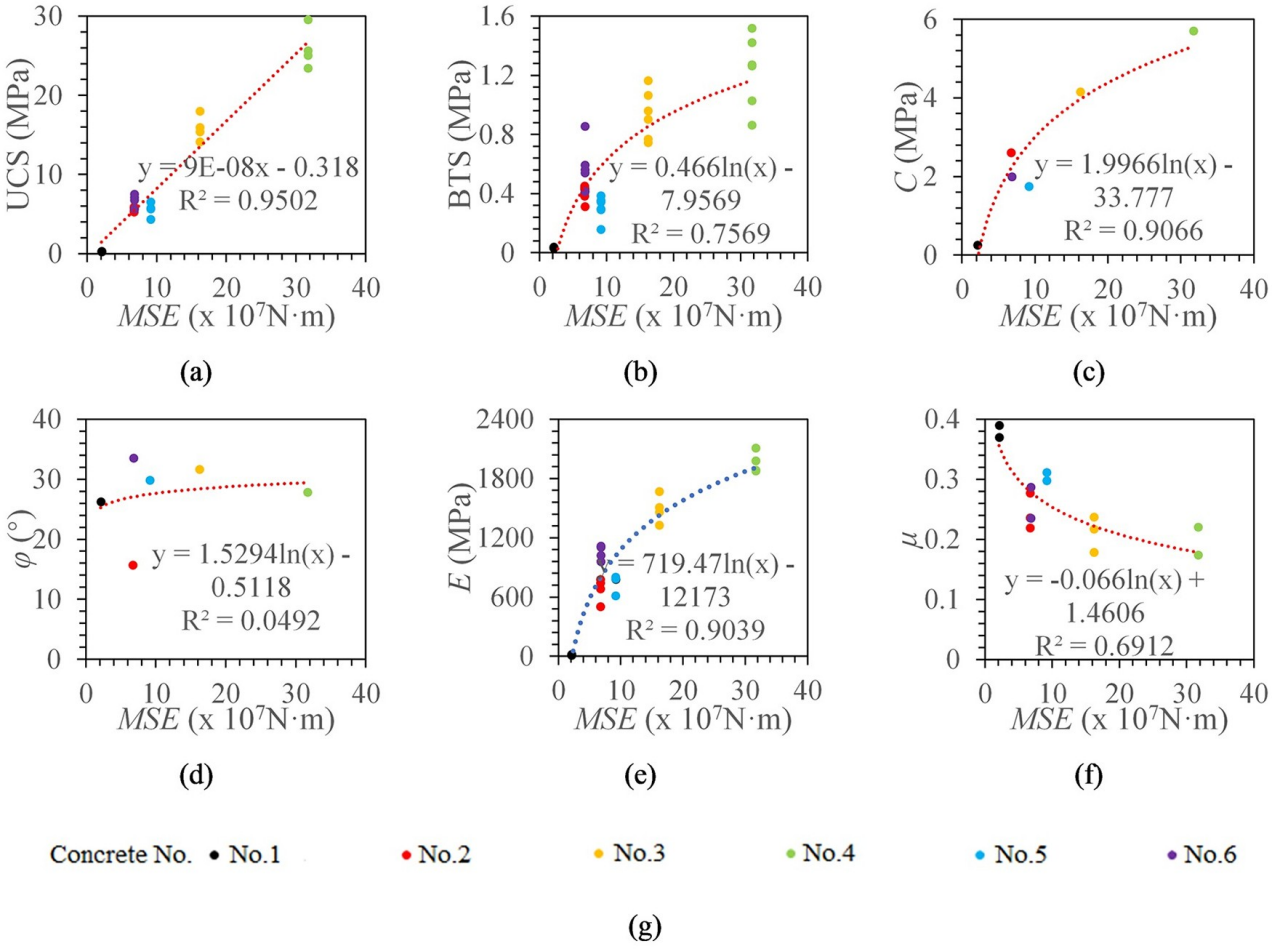
The relationship between MSE and concrete mechanical properties. (a) The relationship between MSE and UCS. (b) The relationship between MSE and BTS. (c) The relationship between MSE and *C*. (d) The relationship between MSE and *φ*. (e) The relationship between MSE and *E*. (f) The relationship between MSE and *μ*. (g) legend.

The MSE of each concrete specimen can be obtained according to Eq (5). It can be seen from Fig 14 that MSE is a linear model with UCS, and a logarithmic model with BTS, cohesion, elastic modulus, and Poisson’s ratio. The correlation coefficients of MSE with UCS, cohesion, and elastic modulus are all above 0.9. The correlation coefficients of MSE with BTS and Poisson’s ratio are 0.7569 and 0.6912, respectively, and there is no significant correlation with the internal friction angle.

## Discussion

### Compared with previous research work

The limitations of this study and the solutions in other literature are as follows: i. In the actual drilling process, water is continuously used to cool the drill bit, the influence of water on the drilling was ignored due to the shallow drilling depth in this experiment. Many devices are equipped with corresponding pumping stations, which can provide flushing water [10, 11]. ii. The specific gravity/density difference of concrete blocks was used to simulate the thrust difference when drilling into different strength concretes, which may be different from the actual situation. Compared with fixing one or two drilling parameters, the method in this paper is closer to the actual situation. To deal with this problem, some scholars have also used roof bolter to conduct experiments in the laboratory [27]. iii. Drilling through the weak seams or bedding hasn’t been implemented in actual coal mines. To address this issue, the displacement sensor was fixed on the roof bolter for data collection in coal mines [26].

Based on the drilling parameters, a lot of research work on prediction methods of rock mechanical properties has been carried out in the laboratory [6–9]. In this paper, the prediction method of rock mechanical properties was extended to the process of borehole drilling. The feature of borehole drilling is upward drilling, and the drilling system is an adaptive system. The drill rod and bit used in this experiment are the drillrod and bit used in the actual borehole drilling. Moreover, the total gravity of the concrete block and the gravity loading plate, instead of the anchor rig thrust, was adopted, which enables upward drilling and adaptive drilling system. This drilling method is closer to the actual situation. Besides, the correlation between borehole drilling parameters and various rock mechanical parameters is analyzed comprehensively. Under the stable drilling conditions, the ROP is determined by the mechanical properties of the rock. For the same homogeneous strata, the ROP only changes when the drilling conditions change. The ultimate goal of this research is to realize the real-time detection of the mechanical properties of the roadway roof strata.

### New findings

First, the drilling process can be divided into an initial stage and a stable stage. From the curves shown in Fig 4, it is evident that the ROP is relatively faster during the first period and then slows down. The initial stage range of concrete with different strength is different, but it is mainly concentrated in 0-30mm section. This phenomenon may be related to the structure of the drill bit and the size of drill cuttings. When the cutting particles are larger, the MSE is smaller, while the drilling efficiency is higher [29]. The broken rock fragments can quickly slide out of the borehole when the drill bit penetrates the concrete at the beginning, which prevents the rock fragments from being broken into smaller fragments to make the ROP significantly higher.

Second, rotation speed and torque are not ideal for predicting rock physical and mechanical properties. Rotation speed and torque are not highly correlated with the concrete mechanical parameters. As shown in Figs 7 and 8, when the concrete specimen strength reaches a certain value and then gradually increases, rotation speed does not continue to decrease, and the torque does not continue to increase. This may be due to the reason that penetration depth per revolution is very small when the concrete specimen strength reaches a certain value, which makes rotation speed increase and torque decrease.

Third, during drilling these boreholes, the torque work ratio accounts for more than 99%, which increases with the increases of rock strength (as shown in Fig 11).

Fourth, the ROP is the best choice for the estimation of rock mechanical parameters. Through the correlation comparison of rock mechanical parameter models, it can be found that there is a weak correlation between borehole drilling parameters and internal friction angle. The optimal prediction models of rock mechanics parameters are presented in Table 4. From Table 4, the correlation between each index and UCS, cohesion and elastic modulus is mainly concentrated in the range of 0.9~1.0; the correlation between each index and BTS is mainly concentrated in the range of 0.8~0.9; the correlation between each index and Poisson’s ratio is mainly concentrated in the range of 0.7~0.8. Compared with ROP, torque work ratio has a slightly higher correlation with UCS and cohesion. Among all indexes, the ROP has the highest correlation with BTS, elastic modulus and Poisson’s ratio. At present, considering that it is difficult to collect borehole drilling parameters, the torque work ratio requires more parameters, so the ROP is the best choice for estimating rock mechanical properties.

**Table 4:** The optimal prediction models of rock mechanics parameters.

Model type	Required parameters	Model expression
UCS prediction model	*V*	*UCS* = 41.662*e*^−1.447*V*^ R² = 0.9631
	*V*, *N*	*UCS* = −12.41 ln(*h*) − 13.256 R² = 0.9575
	*T*, *N*, *V*	*UCS* = 7*E* − 08*W*_*T*_ − 0.1273 R² = 0.9491
	*F*, *T*, *N*, *V*	*UCS* = 8*E* − 233*e*^537.89*η*^ R² = 0.9662
	*F*, *T*, *N*, *V*	*UCS* = 9*E* − 08*MSE* − 0.318 R² = 0.9502
BTS prediction model	*V*	*BTS* = 1.8291*e*^−1.123*V*^ R² = 0.8718
	*F*, *T*, *N*, *V*	*BTS* = 3*E* − 185*e*^425.29*η*^ R² = 0.8546
Cohesion prediction model	*V*	*C* = −2.183 ln(*V*) + 2.9033 R² = 0.9326
	*T*, *N*, *V*	*C* = 2.048 ln(*W*_*T*_) − 35.047 R² = 0.9179
	*F*, *T*, *N*, *V*	*C* = 7*E* − 161*e*^370.66*η*^ R² = 0.9492
	*F*, *T*, *N*, *V*	*C* = 1.9966 ln(*MSE*) − 33.777 R² = 0.9066
Elastic modulus prediction model	*V*	*E* = −779 ln(*V*) + 1051.6 R² = 0.9534
	*T*, *N*, *V*	*E* = 734.5 ln(*W*_*T*_) − 12562 R² = 0.914
	*F*, *T*, *N*, *V*	*E* = 8*E* − 264*e*^614.1η^ R² = 0.9479
	*F*, *T*, *N*, *V*	*E* = 719.47 ln(*MSE*) − 12173 R² = 0.9039
Poisson’s ratio prediction model	*V*	*μ* = 0.0589*V* + 0.1802 R² = 0.7278
	*F*, *T*, *N*, *V*	*μ* = −21.679*η* + 21.872 R² = 0.7208

### Research significance

MWD is an important part of the intelligent development in coal mines. Borehole drilling is a part of the operational cycle, which can be used to detect the characteristics of the roof rock layer in real-time to optimize the support design. However, compared with drilling in petroleum, there is relatively little research on MWD in coal mines. The MWD device was developed based on the borehole drilling process in this article, which not only provides a basis for further research on the application of MWD in coal mines but also provides a new method for rock physical and mechanical parameter testing in the laboratory.

In this paper, borehole drilling parameters like rotation speed, ROP, torque, and SPL were recorded during drilling concrete blocks with various strengths. Furthermore, the response characteristics of each drilling parameter were comprehensively analyzed, which provides a reference for scholars to deeply understand the rock breaking process and the acquisition method of drilling parameters. Besides, the correlation between 4 drilling parameters (*V*, *N*, *T*, *L*_*p*_), 4 composite indexes (*h*, *W*_*T*_, *MSE*, *η*), and 6 rock mechanics parameters (UCS, BTS, *C*, *φ*, *E*, *μ*) was analyzed. Meanwhile, the best fitting model of each drilling index and rock mechanics parameter was obtained. These model coefficients change when drilling with the roof bolter, but the model type does not change. These research conclusions are significant for the intelligent detection of rock formation in coal mines.

### Future research work

For long-distance roadway excavation, drill bits of different sizes may be used to drill boreholes. The drill bit diameter has a greater influence on ROP, which may cause misjudgment of the strength and thickness of the rock formation when the ROP is used as the prediction model. The volume of broken rock per unit time (*V*_t_) may have a strong correlation with rock strength and can be solved as:

$$V_{t}=\pi r^{2}V$$ (7)

The prediction of the strength and thickness of the rock formation based on the volume of broken rock per unit time will be the future research direction.

## Conclusions

In this paper, an attempt was made to extend the application of MWD to predict of mechanical properties of roadway roof strata. The relationship between various drilling indicators and physical and mechanical parameters was analyzed based on the designed MWD device. The study yielded the following conclusions:

1. The drilling process can be divided into the initial stage and stable stage. The ROP is significantly higher at the initial stage (0-30mm), which avoids misjudging the initial stage and stable stage as two distinct rock formations.
2. Torque and rotation speed are not ideal choices for estimating rock physical and mechanical parameters. There is a UCS critical value so that the rotation speed increases and the torque decreases with the UCS increases when the UCS is greater than the critical value.
3. The torque work ratio and the penetration depth per revolution are significantly related to UCS, BTS, cohesion, and elastic modulus. The torque work ratio accounts for more than 99%, and it increases with the rock strength increases.
4. At present, the ROP is the best choice for estimating rock mechanical parameters. There is a significant correlation between ROP and UCS, cohesion and elastic modulus (R^2^ > 0.9). The ROP is highly correlated with BTS (R^2^ = 0.8718). There is a moderate correlation between ROP and Poisson’s ratio (R^2^ = 0.7278). There is no correlation between ROP and internal friction angle (R^2^ = 0.1094).

## Supporting information

S1 Data (ZIP)
